# Advances in Pancreatic Islet Transplantation Sites for the Treatment of Diabetes

**DOI:** 10.3389/fendo.2021.732431

**Published:** 2021-09-13

**Authors:** Fritz Cayabyab, Lina R. Nih, Eiji Yoshihara

**Affiliations:** ^1^Lundquist Institute for Biomedical Innovation at Harbor-UCLA Medical Center, Torrance, CA, United States; ^2^David Geffen School of Medicine at University of California, Los Angeles, CA, United States

**Keywords:** islet transplantation, diabetes, vascularization, biomaterials, stem cells

## Abstract

Diabetes is a complex disease that affects over 400 million people worldwide. The life-long insulin injections and continuous blood glucose monitoring required in type 1 diabetes (T1D) represent a tremendous clinical and economic burdens that urges the need for a medical solution. Pancreatic islet transplantation holds great promise in the treatment of T1D; however, the difficulty in regulating post-transplantation immune reactions to avoid both allogenic and autoimmune graft rejection represent a bottleneck in the field of islet transplantation. Cell replacement strategies have been performed in hepatic, intramuscular, omentum, and subcutaneous sites, and have been performed in both animal models and human patients. However more optimal transplantation sites and methods of improving islet graft survival are needed to successfully translate these studies to a clinical relevant therapy. In this review, we summarize the current progress in the field as well as methods and sites of islet transplantation, including stem cell-derived functional human islets. We also discuss the contribution of immune cells, vessel formation, extracellular matrix, and nutritional supply on islet graft survival. Developing new transplantation sites with emerging technologies to improve islet graft survival and simplify immune regulation will greatly benefit the future success of islet cell therapy in the treatment of diabetes.

## Introduction

Diabetes is a complex metabolic disease in which the body’s ability to produce or respond to insulin is impaired, resulting in hyperglycemia. To date, approximately 451 million people worldwide have diabetes, and the World Health Organization (WHO) projects this number to increase to 693 million by 2045 (1). The emergence and progression of both autoimmune-induced type 1 diabetes (T1D) and stress-induced type 2 diabetes (T2D) are affected by various genetic, metabolic, environmental, and immune factors. Nevertheless, the failure of islet β cells mass or function is considered a predominant factor that impacts the pathology of diabetes. While, the pancreas is thought to be the main organ affected, both T1D and T2D and their associated complications involve multiple organs with heterogenous pathogenic mechanisms. Diabetes is attributed to defects in insulin secretion and action; disturbance in carbohydrate, fat, and protein metabolism; faulty micro- and macro-vascularization; chronic inflammatory state. All these pathologies result in complications, such as of blindness, retinopathy, nephropathy, neuropathy, and cardiovascular diseases (2–9). Emerging evidence suggests that diabetes is a risk factor for various other diseases, as illustrated by SARS-CoV-2 infection complications resulting from the direct infection in the endocrine and exocrine pancreas (10–30). The global burden of diabetes is steadily rising, affecting every nation and population. Strategies for mitigation, control, and treatment of diabetes have been the subject of intense research. Daily insulin injection therapy remains to be the standard care for patients with T1D, late-stage of T2D, and in some rare forms of diabetes (31). However, a daily insulin injection therapy, while life-saving, does not exactly recapitulate the effectiveness of endogenous control of blood glucose by β cells. In addition, the insulin injection therapy represents a chronic and costly burden for diabetic patients, and it does not entirely eliminate the risk of acute and chronic complications related to diabetes. Pancreatic islet transplantation, in which pancreatic islets are isolated from donors and percutaneously infused into the liver *via* the portal vein, is a current treatment for insulin-dependent diabetes (32–37). This procedure has been performed successfully on patients with T1D, providing exogenous insulin independence for several years. Additionally, pancreatic islet transplantation can be superior to daily insulin therapy in delaying diabetes-related complications and in exerting overall metabolic control (34, 37, 38). Despite its efficacy, this allograft islet transplantation cannot be universally performed because of shortages in islet donors and side effects associated with the life-long need for immunosuppression.

Recent advancements in the field of stem cells have brought us closer to addressing the shortage of cadaveric islets for use in transplantation. Various protocols utilizing human embryonic stem cells (hESCs) or human induced pluripotent stem cells (hiPSCs) have been developed to differentiate these cells into β-like cells with key markers for mature pancreatic islets (39–45) (31). These stem cell-derived β-like cells are capable of sensing blood glucose levels and are also capable of secreting various levels of insulin when transplanted into animal models of diabetes. Thus, these strategies can provide an alternative source for insulin-producing β-like cells derived from stem cells. Despite their therapeutic potential, the clinical viability of transplanting stem cell derived insulin-producing cells poses many challenges including the optimization of differentiation and maturation (46–48), graft rejection induced by one’s immune system and long-term survival *in vivo* (49–52). Although, the current transplantation location used in clinical settings is through the hepatic portal vein in the liver, there is a growing consensus that the hepatic milieu may not be hospitable for functional islet transplantation and their long-term viability, not only for cadaveric human islets (53) but also stem cell derived islets. Therefore, it is important to improve the efficacy of islet transplantation by the development of biomaterials and transplantation sites which enhances graft survival for the future advances on islet therapy in diabetes.

In this review, we summarize current advances made on islet transplantation sites and how they affect graft rejection, immune response, and vessel formation.

## Transplantation Sites

The pancreatic islet transplantation procedure involves isolating pancreatic islets capable of secreting insulin from an autologous source, such as donors, or from the autonomous sources (a total pancreas resection caused by pancreatitis or similar injury). Pancreatic islets are released from the pancreas *via* a combination of chemical methods that involve collagenase and neutral protease digestion (54, 55) and mechanical methods (56). Then, the islets are purified through variable centrifugations to separate islets from the pancreatic acinar and ductal tissue (57, 58). The isolated and purified pancreatic islets are then transplanted into the liver by percutaneous transhepatic islet transplantation at the portal vein sites. The liver is currently the preferred transplantation site because the procedure is minimally invasive with ease of access and has low rates of bleeding and thrombosis.

The liver can also provide oxygenation to the transplanted islets *via* the portal circulation until revascularization occurs. In addition, this approach allows insulin to be delivered to the liver and intestines. In 2000, Shapiro et al. reported the first proof of concept for pancreatic islet transplantation into the liver of seven T1D patients (35). A *post hoc* analysis of autologous islet transplantation study was reported that 173 pancreatectomized patients with autologous transplantation and 262 diabetes patients with allogeneic transplant showed that 85% of autologous transplant recipients and 66% of recipients of allogeneic transplant recipients were insulin-independent for two years after surgery (57).

**Figure 1:**
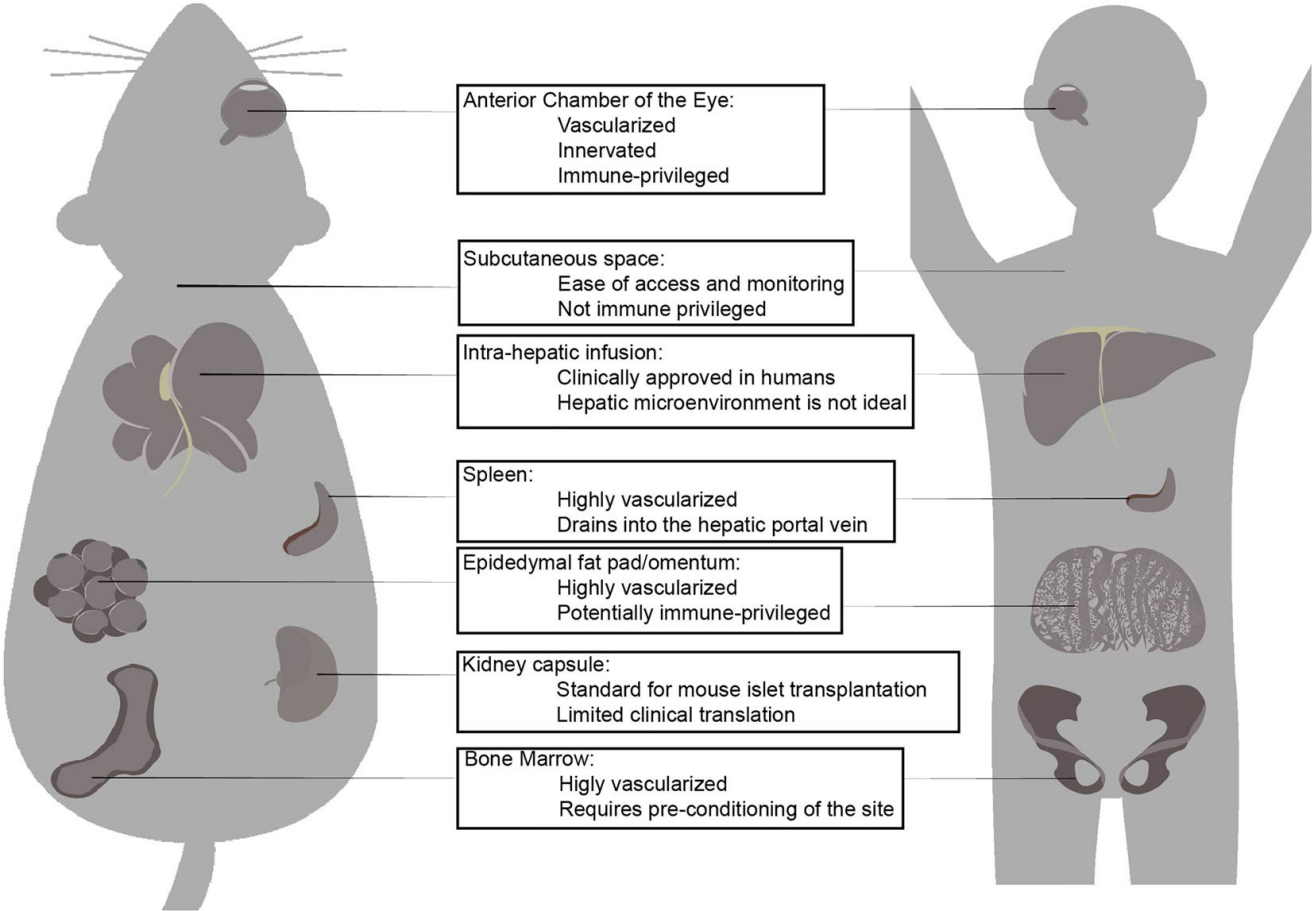
Islet transplantation sites that have been tested in mouse models and higher mammalian models. The three major factors contribute to success of islet transplantation are the 1. Presence of vascularization and innervation, 2. Immune-modulating factors which affect innate inflammatory response and graft rejection, 3. Accessibility for surgical procedure and absence of major surgical complications. Each transplantation site shows advantages and disadvantages which have been explored. While intra-hepatic infusion is the only clinically applied site for islet transplantation, there are extra-hepatic candidate sites that may superior islet transplantation site. The anterior chamber of the eye is highly vascularized innervated and immune-privileged in most conditions low and atypical expression of MHC class I and II, as well as presence of anti-inflammatory and immune-modulating factors in the intra-ocular fluid is beneficial for islet engraftment. Subcutaneous space is not immune-privileged and poorly vascularized but ease of access and simplicity of surgical procedure and complications makes it an attractive site for islet transplantation. Liver *via* hepatic portal infusion is the only clinically approved site of transplantation due to success of Edmonton protocol, but extensive loss of islet necessitates for multiple islet donors. Hepatic micro-environment is considered a factor in long-term decline of viability of transplanted islets. Spleen is highly vascularized and drains into the hepatic portal vein and may contain immune-modulating Tregs. Omentum or epididymal fat (murine equivalent) is highly vascularized, and potentially-immune privileged. It can accommodate large islet volume, including a different immuno-modulating co-transplanted cells and biomaterials and devices. Kidney capsule is routinely used as site for islet transplantation in murine subjects, but clinical translation to humans is limited due to common diabetes-related renal complications. Bone marrow is highly vascularized but requires pre-conditioning before it can be considered for islet transplantation.

Islet transplantation has proven to be successful in controlling hyperglycemia and providing insulin-independence for many diabetic patients. However, more research is needed to improve its success rate for long-term application. A key component in successfully optimizing islet graft survival after transplantation is the rapid establishment of blood flow for nutritional supply, oxygen supply, and immune regulation. It is estimated that approximately 50% of transplanted islets survive in the first few days of transplantation because of instant blood-mediated inflammatory reaction (IBMIR) and acute immune response (59, 60). Fewer transplanted islet survives thereafter because of the lack of vascularization for oxygen and nutritional supply (61–63). This loss of freshly transplanted pancreatic islets requires the need for two or more donors for each recipient of pancreatic islets. In the long-term, transplanted pancreatic islets decline in function, possibly due to metabolic exhaustion or an inhospitable transplantation microenvironment. Additionally, graft rejection can occur because of the innate and adaptive immune response, which requires the need for life-long administration of immunosuppressants (64). It is still unclear how the transplantation site microenvironment affects the survival and functionality of transplanted pancreatic islets beyond the need for sufficient vasculature. While the liver is historically the preferred site, research indicates that the hepatic microenvironment is not as hospitable to transplanted pancreatic islets as was initially thought. Thus, researchers are investigating different transplantation sites to identify more hospitable and optimal locations for islet transplantation. In the following section, we summarize the different transplantation sites being explored for islet transplantation (**Figure 1**). Current advances on islet transplantation in these differential sites are also summarized (**Table 1**).

**Table 1:** Summary of Transplantation Sites, Biomaterials and Resident immune cells.

Alternative Site	Islet Used	Recipient Species	Number of Islets per recipient	Description	Results	Reference
Anterior Chamber of the Eye (ACE)	Islets from PdxCreER-GCAMP in C57BL/6N background	C57BL/6N albino mice	~300 IEQ	Development of a non-invasive *in vivo* fluorescence imaging of islets using the anterior chamber of the eye as a natural window	Normoglycemia observed at ~2 weeks after transplantation, monitored for approximately over 200 days	(65)
	Islets from C57BL/6 and 129X1 mice	Streptozotocin-induced diabetic C57BL/6 mice and Nude-Foxn1nu (nude) mice	~300 IEQ	Study determining the involvement of cholinergic innervation in insulin secretion function of islets	Not available: metabolic effect on daily blood glucose change was not investigated	(66)
	Islets from C57BL/6J and Tie2-GFP mice	Streptozotocin-induced diabetic athymic male nude mice (B6;Cg/JBomtac-Foxn1^nu^N3)	~150-200 IEQ	Determination of the contribution of donor endothelial cells present from isolated islet in revascularization process	Normoglycemia reached in Median of 12.5 days (fresh islets) or median of 7 days (after 4-day cultured)	(67)
	Islets from C57BL/6 mice and NOD mice	Streptozotocin-induced diabetic C57BL/6 mice; NOD-SCID mice given diabetogenic splenocytes	~25-125 IEQ	Evaluation and real-time visualization of how autoimmunity can occur during T1D	Normoglycemia reached in approximately 12 days, and observed until 47 days	(68)
	Mouse pseudo-islets from Ins1(Cre) knock-in in C57BL/6J background	Streptozotocin-induced diabetic C57NL/6J mice	~100 pseudo-islet approximately	Proof of concept study of increasing transfection efficiency in beta islet by de-aggregating beta islets and then transfecting with adeno-virus before allowing to re-aggregate into pseudo-islet before transplantation in anterior chamber of the eye	Normoglycemia was achieved within two weeks in unreported percentage of mice, and maintained for approximately 40 days	(69)
	Mouse islets from 2 month-old and 18 month-old C57BL/6 male mice; human islets from non-diabetic donors	Streptozotocin-induced C57BL/6 mice	~200 IEQ	A study investigating the effect of age-dependent impairment in islet function and vascularization.	Approximately 50% of diabetic recipient achieved normoglycemia within 50 days after transplantation, with majority maintaining glycemic control for up to 11 months	(70)
	Allogeneic islets from baboon model of diabetes	Allogeneic transplantation into a Streptozotocin-induced diabetic baboon	~18,000 to 20,000 IEQ (approximately 2100 IEQ/Kg)	Study evaluating feasibility of ACE as islet transplantation site in pre-clinical model of streptozotocin-induced diabetes	Exogenous insulin requirement decreased after 3 months, monitored until 357days	(71)
	Allogeneic islets from a healthy cynomolgus monkey	Allogeneic transplantation into the T2D cynomolgus monkey	~12,000 IEQ (1500 IEQ/Kg)	Evaluation of ACE as transplantation site for islet pre-clinical model of high-fat diet induced diabetes	Normoglycemia post-transplantation (88measurements); Observed for 348 days post-transplantation (POD 97-POD185; iridectomy POD186-POD348)	(72)
Bone Marrow	Syngeneic islets from C57BL/6 mice	Streptozotocin-induced diabetic C57BL/6 mice	~125-500 IEQ	Proof of concept study of transplanting islet into an easily accessible bone marrow	52% of mice transplanted with 125 IEQ achieved normoglycemia within 85 days (median), and 76% of mice transplanted with 250 IE achieved normoglycemia within 14 days (median)	(73)
	Autologous Islet transplantation in humans	Four patients with contraindication for intraportal islet infusion	~32,000 to 334,600 total IEQ per patient (666-4,780 IEQ)	Pilot clinical study of testing feasibility and safety of autologous human islet transplantation into the patients’ own bone marrow		(74)
	Allogeneic human islet	Patients with T1D and contraindication for intraportal islet infusion, and T1D patients in randomized trial	~287,000 to 1,125,116 total IEQ (2,727 – 10,684 IEQ/Kg)	Feasibility study and pilot randomized trial of patients with T1D to study safety and outcome of allogeneic islet transplantation into the bone marrow		(75)
	Islets from C57BL/6 or BALB/c mice6	Alloxan-induced diabetic C57BL6 mice	450 IEQ	Study that evaluated bone-marrow precondition *via* local irradiation to improve syngeneic and allogeneic transplantation in mice	Normoglycemia reached within 1 week post-transplantation and monitored for up to 6 weeks post-transplantation	(76)
Subcutaneous	Landrace pig islets with pig bone marrow-derived mesenchymal stem cells (BMMSC), pig adipose-derived mesenchymal stem cells (AMSC)	Streptozotocin-induced diabetic Wistar rats and cynomolgus monkeys	125-300 IEQ/g for Wistar rat recipients, 15,000 to 62,500 IEQ/Kg for primate recipients	Porcine islets and mesenchymal stem cells from bone marrow and adipose tissue, co-encapsulated in P.E. alginate coated, collagen matrix device were transplanted subcutaneously in Wistar rats and cynomolgus monkeys to determine if this system could improve vascularization, implant oxygenation, and metabolic control in short term and long term.	For islets alone, mean weeks of normoglycemia is 28 weeks, while Islets with adipose-derived MSC and bone marrow-derived MSC have 23 weeks and 30 weeks, respectively. Co-encapsulation of MSC did not improve long-term viability	(77)
	Syngeneic islets from C57BL/6 mice, or BALB/c mice, or human islets	Streptozotocin-induced diabetic C57BL/6 mice, and Rag^-/-^ mice	~500 IEQ (syngeneic transplant), ~2000 human islet IEQ in immunodeficient mice	The subcutaneous space was pre-treated with medically-approved nylon or silicone catheter for one month before being removed (device-less). One month implantation was enough to induce a subcutaneous space with local neovascularization without formation of thick, mature fibrotic scar, before being transplantation with islet	91% of diabetic mice reversed diabetes by day 60 and maintained normoglycemia for more than 100 days	(78)
	Human embryonic stem cell-derived pancreatic endoderm	Streptozotocin-induced diabetic, immunodeficient B6/Rag^-/-^ mice	0.5-1.0 x 10^7^ cells	Use of similar ‘device-less” technique of implanting nylon catheter in subcutaneous space for month before removal. Subsequent implantation of human embryonic stem cell-derived pancreatic endoderm for *in vivo* maturation	Of the mice that were transplanted at epididymal fat and un-treated subcutaneous space, only 33% achieved normoglycemia. For mice that were transplanted in ‘device-less’ subcutaneous space, 100% achieved normoglycemia for an average of 99.8 ± 3.8 days	(79)
	Wistar rat islets	Streptozotocin-induced diabetic SCID/beige mice and immune-suppressed Sprague-Dawley Rats	~750 IEQ	Islets were embedded in submillimeter collagen cylinders, coated with endothelial cells before being transplanted subcutaneously.	five out of six diabetic rats have restored normoglycemia within 10 days for 21 days	(80)
	C57BL/6 syngeneic islets, and human islets	Streptozotocin-induced diabetic C57BL/6 and NOD/SCID mice	~100-400 mouse IEQ and ~2000 to 2500 human IEQ	Transplantation into a specific subcutaneous space in the inguinal subcutaneous white adipose tissue allowed for neovascularization and connection with feeding vessels from the inferior epigastric artery and vein.	Six out of eight diabetic mice receiving both CTLA4 Ig and anti-CD40L antibody regained normoglycemia within approximately 60 days, compared to one out of five/six of mice receiving either anti-CD40L or CTLA4Ig only. Normoglycemia was maintained for a duration of approximately 120 days after which the graft was removed	(81)
	F344 rat islet, Porcine islets	SCID mice, total-pancreatectomized pig as model for T1D	1,000-2,000 rat islets; approximately 7,007 to 10,005 IEQ/Kg of porcine islets was used for porcine recipients	Adipose-derived mesenchymal stem cell sheet seeded with islet was transplanted subcutaneously in SCID mice and total-pancreatectomized pigs	100% of SCID mice transplanted with four islet-MSC sheet achieved normoglycemia within 1 week, and maintained normoglycemia for 84 days; normoglycemia was achieved in one week, and maintained until second week during which graftectomy was performed	(82, 83)
	Sprague-Dawley rat islets	diabetic athymic BALB/c-nude mice	~4,000 rat IEQ and ~8,000 human IEQ	Esterified collagen scaffold functionalized with heparin contained varying number human adipose-derived stem cells and islets, transplanted subcutaneously	Higher number of human adipose-derived stem cells resulted in normoglycemia within 1 day post-transplantation, and maintained for up to 100 days	(84)
	Murine, porcine and human islets and cynomolgus islets (auto-transplantation)	Streptozotocin-induced diabetic immune-incompetent B6/SCID and Balb/c/nude mice and immune-competent wild-type B6 mice (with immune-suppressive regimen), as well as 90%-pancreatectomized cynomolgus monkey	~400-800 murine IEQ; 250-500 porcine IEQ; 400 human IEQ	Pancreatic islets were transplanted subcutaneously with a viability matrix containing collagen, L-glutamine, FBS, and sodium bicarbonate and media that affected long-term functionality of engrafted islets	Normoglycemia was achieved within 24 hours post-transplantation with graft survival and maintained for up to 127 days (immune-incompetent mice) and up to 529 days (wild-type mice under immune-suppressive regimen); T1D cynomolgus monkey (auto-transplantation) maintained normoglycemia until 820 days post-transplantation.	(85)
	Pseudo-islets from de-aggregated rat or human islets cells	Streptozotocin-induced diabetic SCID/bg mice	1.5 x 10^6^ islet cells (dose equivalent of approximately 750 IEQ)	Collagen gel scaffold embedded with pseudo-islets with modifiable proportion of the different cell types of the pancreatic islet for uniform pseudo-islet size, cell composition and proportion	Six out of nine SCID/B6 mice transplanted with pseudo-islets and adMSC returned to normoglycemia in approximately 10 days until day 21, during which graftectomy was performed	(86)
	C57BL/6 and BALB/c mouse islets, Sprague-Dawley rat islets, human islets, HUES8 stem-cell derived β cell clusters.	Streptozotocin-induced diabetic male C57BL/6, male SCID-beige mice, and male NOD/NSG mice and healthy beagle dogs	~600-700 IEQ ~1700 human islet clusters, ~2500 HUES8 stem-cell derived β cell clusters	Use of medical-grade, silicone-polycarbonate-urethane biomaterial encapsulating an alginate core containing either syngeneic, allogeneic, or xenogeneic rodent islets, or human islets or HUES8 stem-cell derived β cell clusters to assess foreign body reaction, immune-protective function, and normoglycemia function	Syngeneic mouse model achieved normoglycemia within one week for 120 days (13 out of 17 mice), while normoglycemia was achieved within one week for up to 100 days in immune-deficient mouse model (8 out of 11 mice). In immune-competent mice, normoglycemia was achieved within a week for up to 8 weeks (10 out of 16 mice)	(87)
	Sprague-Dawley male rats	Streptozotocin-induced diabetic male C57BL/6 mice and female Gö;ttingen minipigs	500 rat IEQ; 1500 rat IEQ	Proof of concept study using an inverse breathing encapsulation device (iBED) that supplies oxygen to transplanted islet *via* gas-solid reaction between LiO_2_ and cellular metabolism bi-product CO_2_.	Improved iBED version resulted to eight out of ten C57BL/6 mice achieving normoglycemia for approximately 92 days, with better glucose metabolism even after 90 days. Non-fasting blood glucose level was not reported in minipigs, but retrieved device exhibited more surviving islets than controls after 1 and 2 months of implantation	(88)
Omentum	Syngeneic C57BL/6J female mouse islets	Streptozotocin-induced diabetic C57BL/J6 male mice and streptozotocin-induced NOD-SCID male mice	~600 mouse IEQ	Study comparing three leading extra-hepatic islet transplantation site (subcutaneous, small bowel mesentery, epididymal fat pad) for synthetic vasculogenic hydrogel-based islet transplantation	The islets with the vasculogenic hydrogel transplanted into the epididymal fat pad achieved normoglycemia within two weeks, for more than 35 days up to 100 days (approximately 75% of recipients)	(89)
	Lewis male rat islet	Lewis female rats	~10,000 IEQ/Kg	Non-biodegradable knitted polymer mesh inserted into the omentum with subcutaneous access for 4 weeks before islets are introduced. Insulin pellets were also introduced into the Lewis rat recipients	All ten rat islet recipients achieved blood glucose of 8mmol/L as a result of combined insulin pellet and transplanted islet, and maintained for up to 100 days	(90)
	Wistar Furth male rat islets, Cynomolgus monkeys	Streptozotocin-induced diabetic female Lewis rats, Streptozotocin-induced diabetic cynomolgus monkeys	17,338 ± 881 IEQ/Kg for syngeneic rat model; ~3000 IEQ of allogeneic rat model; Cynomolgus monkey islet of approximately 48,700 IEQ (9347 cynomolgus monkey IEQ/Kg)	Islet transplantation into the omentum utilizing a biological, resorbable plasma-thrombin scaffold to monitor metabolic improvement in diabetic rats, as well as cytoarchitecture of transplanted islets	Seven out of seven syngeneis rats achieved normoglycemia within 2 days and maintained normoglycemia for more than 200 days. Four out of four immune-suppressed allogeneic rats achieved normoglycemia five days post-transplantation, and maintained for more than five weeks.	(91)
	Cynomolgus monkey islets	Non-diabetic cynomolgus monkeys; Streptozotocin-induced diabetic C57BL/6 mice	Approximately 1500 cynomolgus monkey islet in intraperitoneal space of diabetic C57BL/6 mice; approximately 5000 cynomolgus monkey islets were seeded in 5mL of alginate formulation at seeding density of 1000 islets per mL of alginate formulation	Study investigating foreign body response to different immune-modulating formulation of alginate in islet encapsulation method, as tested and transplanted in non-diabetic non-human primates. Islet viability is measured after 1 month and 4 months of transplantation into the omental bursa.	Alginate formulation SLG20 allowed for normoglycemia in diabetic C57BL/6 mice for approximately 120 days without the need for immunosuppressant. Marginal fibrosis was observed after 1 month of transplantation in these C57BL/6 mice. The same results were not recapitulated when the same formulation was tested in cynomolgus monkeys; Instead, a different alginate formulation, Z1-Y15, showed reduced foreign body response in the form of fibrosis when tested in non-human primates. Six out of seven encapsulated islets showed higher viability after retrieval from transplantation. Blood glucose control was not investigated in these non-human primates.	(92)
Spleen	Islets from Pancreatectomized Mongrel dogs	Autotransplantation Pancreatectomized non-diabetic Mongrel dogs	Approximately 9000-13,000 IEQ per recipient	Comparative study of omental pouch *vs* splenic site for islet transplantation with focus on hypoglycemic correctional response in these animals	Time to normoglycemia was not indicated but beta islet response to insulin-induced hypoglycemia was deemed normal while alpha cell response was not. The response in omentum and splenic transplantation were similar	(93)
	Syngenetic islets from C57BL/6	Streptozotocin-induced diabetic C57BL/6 mice (Syngeneic)	Approximately 50-200 IEQ per recipient	Comparative study of hepatic portal vein, kidney capsule and spleen as islet transplantation site	Spleen has the lowest number of islets required to achieve normoglycemia, compared to portal vein or kidney with reduced inflammation and potential expansion of islet graft.	(94)
	Porcine Islets from fetal pigs	Adult pancreatectomized Westran pigs	Approximately more than 5000 IEQ per recipient	Comparative study of kidney capsule, hepatic portal vein and spleen as islet transplantation site	Normoglycemia was achieved by day 120 post-transplantation. Glucose metabolism is better in kidney than liver and spleen.	(95)
	Human islets or C57BL/6 murine islets	Alloxan-induced diabetic C57BL6 mice (syngeneic) or RAG-1 or SCID immunodeficient mice (recipient for human islets)	Approximately 2000 IEQ per recipient	Comparative study of Portal vein, Quadricep muscles, kidney capsule, liver capsule, and splenic capsule as islet transplantation site	Spleen and liver capsule were inferior compared to other transplantation site. Skeletal muscle and portal vein should similar engraftment efficiency while kidney capsule performed yielded the best outcome at 75% and 100% success rate for human and murine islet transplantation	(96)
	Syngeneic islets from C57BL/6 mice	Streptozotocin-induced diabetic C57BL/6 mice (syngeneic)	Approximately 300 IEQ per recipient	Comparative study of hepatic sinus tract *vs* Splenic parenchyma as islet transplantation site in syngeneic murine models of islet transplantation	Spleen performed better than hepatic sinus tract as islet transplantation site in term of glucose metabolism. Normoglycemia was observed by day 10 post-transplantation for both sites	(97)
Intramuscular	Porcine islets from Lardrace large white pigs	Pancreatectomized baboons (Papio anubis)	Approximately 10,000 IEQ/Kg per recipient	Comparative study of the different immunosuppressive regimen for islet survival in both intraportal vein and muscle (sternomastoid muscle) transplantation site for non-human primates	Normoglycemia was achieved within 24 hours post-transplantation, and the different immunosuppressive regimens allowed for porcine islets to survive beyond 14 days in non-human primate recipients.	(98)
	Human islet	7-year old patient (auto-transplantation)	Approximately 163,000 IEQ (6400 IEQ/Kg)	Clinical study for auto-transplantation of islets into the brachioradialis forearm muscle for a patient with contraindication for intraportal islet transplantation	Observational period lasted for two years, and the patient achieved better quality of life but insulin-independence was not achieved	(99)
	Syngeneic islets from male Lewis rats	Streptozotocin-induced diabetic Lewis Rats (syngeneic)	Approximately 2400 IEQ per recipient	Diabetic rats were pre-treated intramuscularly with biocompatible angiogenic scaffold before islets are transplanted into the abdominal muscle	Islet engraftment was better in bio-compatible scaffold pre-treated rats, with 2-4 times increase in vascularization after 60 days of observation. Normoglycemia was achieved in this cohort I less than 10 days and maintained for up to 60 days	(100)
	Syngeneic islets from Lewis rats	Streptozotocin-induced diabetic Lewis rats (syngeneic)	Approximately 1500-2000 IEQ per recipient	A study developing a reproducible technique for islet transplantation into the bicep femoris of rat models of islet transplantation	Normoglycemia was achieved in less than 20 days and maintained for more than 100 days. It was determined that there is volume-dependent increase in muscle inflammatory response and peri-islet fibrosis. Pearl-on-string transplantation technique allowed for better islet engraftment into the muscle	(101)
	Syngeneic islets from C57BL/6 mice	Streptozotocin-induced diabetic C57BL/6 mice	100-600 IEQ per recipient	Comparative study of different islet transplantation sites, determining marginal mass required and mean time to achieve normoglycemia in murine models of islet transplantation	Kidney required the least number of islets required to achieve normoglycemia followed by omentum and last, liver and muscle. Transplantation in muscle took the longest to achieve normoglycemia compared to other transplantation sites	(102)
	Islets from minipigs	Pancreatectomized minipigs (Non-syngeneic)	Approximately 1000 IEQ/Kg per recipient	Transplantation study into the gracilius muscle in minipig models to determine the best surgical technique to allow for islet engraftment into the muscles	Despite minimizing damage to the muscle during transplantation procedure to minimize immune response, islets transplanted into the muscle did not perform better than islets transplanted into the hepatic portal vein.	(103)
	Neonatal Porcine Islets from 2-5 day old hybrid German landrace piglets	Streptozotocin-induced diabetic NOD-SCID IL2rγ-/- (NSG) mice	Approximately 2500-3000 neonatal porcine islet-like clusters	Comparison of kidney capsule *vs* lower hind-limb muscle as transplantation site for xenogeneic transplantation murine models	Neonatal porcine islet-like clusters needed an *in vivo* maturation period; therefore normoglycemia was achieved after more than 50 days in 50% of the animals transplanted in kidney or muscle. Transplantation into the kidney capsule achieved normoglycemia faster than muscle	(104)
	Islets from Balb/c mice	Streptozotocin-induced diabetic Balb/c mice	Approximately 100-500 IEQ	Comparative study to determine the effects of transplanting islet with Matrigel on engraftment efficiency in femoral muscles	Islet imbedding into Matrigel improved engraftment efficiency into the muscle. The difference in the amount of growth factor present in the Matrigel allowed for difference in engraftment efficiency after day 7 post-transplantation. The proportion of mice achieving normoglycemia following intramuscular transplantation with islets and Matrigel was equal to or greater than mice receiving intraportal transplanted islets	(105)
	Islets from C57BL/6 mice	Alloxan-induced diabetic C57BL/6 nu/nu mice	Approximately 200 IEQ	Study utilizing co-transplantation of islets and polymerized bovine hemoglobulin into abdominal muscle of murine models of transplantation	The polymerized bovine hemoglobulin acts as oxygen carrier that reduced hypoxia in transplanted islet resulting in better engraftment into the muscle	(106)
	Human islets from cadaveric donors	Four patients (allo-transplantation)	Approximately 240-471 IEQ/kg per recipient	Clinical study determining vascularization and function of islets transplanted into the brachioradialis forearm muscle in patients (allo-transplantation)	In three out of four patients, there was a rapid and progressive disappearance of function of the transplanted islets, possibly due to low IEQ transplanted	(107)
	Islets from Lewis rats	Streptozotocin-induced diabetic Lewis rate	Approximately 3000 IEQ	Study determining the suitability of denervated gastrocnemius muscle flap as an islet transplantation site	Because muscle contraction can limit blood flow into intramuscularly transplanted islet, denervation of muscle prior to islet transplantation can improve islet functionality. Blood glucose levels were lower in denervated muscle flap group compared to non-denervated muscle group.	(108)

### Liver Transplantation Site

The liver and portal vein have been identified as the preferred site for islet transplantation because of its ease of accessibility and associated low morbidity. This was evidenced by the fact that most of clinical islet transplantations have been performed at this site. A 1972 study in rats transplanted with syngeneic pancreatic islets into the kidney capsule with venous drainage shunted to the hepatic portal vein achieved complete reversal of hyperglycemia. This study established the importance of transplanting pancreatic islets close to the liver portal circulation to increase the concentration of insulin reaching the liver (109), suggesting that insulin delivery *via* the portal vein is more effective than intraperitoneal infusion. In 2006, Shapiro et al. (110) reported the results of a clinical trial using the Edmonton protocol, a highly improved allogeneic pancreatic islet transplantation protocol developed in 2000 (35). Seven diabetic patients were infused with over 4,000 islet [11,547 +/-1604 islet equivalent (IEQ)] per kg of recipient’s body weight *via* the portal vein, with each recipient receiving the pancreatic islets from two to three brain-dead donors. Seven out of seven of these patients achieved insulin independence in their first year. In 2006, a multicenter clinical trial for pancreatic islet transplantation reported that 21 out of 36 patients achieved exogenous insulin independence for a year, and 16 patients were exogenous insulin independent for two years. The Edmonton protocol has become the foundation for many islet transplantation approaches (110). Improvements in both isolation protocol and standardization protocols now allows islet transplantation from donors to patients to be successfully performed within 72 hours. During the first few days after transplantation, the islets are only oxygenated only *via* diffusion in the low-oxygen tension portal vein. It takes approximately 7 to 14 days before the islet develops a functional vasculature (111), but even then, the transplanted islets chronically maintain a low endogenous oxygen tension compared to native islets (112). As such, transplanted islets suffer hypoxic insults, which contributes to the failure in islet survival (113, 114). Pre-treatment of islets with anti-hypoxic agents before transplantation shows promise in improvement of the islet survive in the short term, but the need for extensive vascularization is still a major issue for the long-term islet survival. Alternative transplantation sites that are pre-existing, or capable of developing extensive vascularization leading to similar nutritional and oxygen supply to the native islets are actively being investigated. Strategies to promote angiogenesis early after transplantation *via* angiogenic biomaterials and factors are also being explored. When islets are transplanted *via* the portal vein, they spontaneously settle into the peripheral branches. This produce risk of portal vein thrombosis and other vascular complications, such as variceal bleeding, intravascular coagulation, and intraperitoneal hemorrhage. While the risk for complete thrombosis is rare, partial thrombosis can occur more frequently and affect the function of both transplanted islets and liver, leading to partial liver damage and necrosis (115). Thrombosis also induces an inflammatory response that is detrimental to long term islet survival. Additionally, it can cause instant blood-mediated immune responses and activate the complement and coagulation cascades, leading to macroscopic clots that can further interrupt blood flow and exacerbate hypoxic insults to the transplanted islets and liver.

Immune responses are exaggerated by MHC mismatch meaning that the source of pancreatic islets have a great impact on both short and long-term islet viability and functionality. If the source of islet transplant is from MHC mismatched another individual or donors, it may require for the use of immune-suppressive regimen in order to delay or avoid graft rejection and extend the long-term viability of transplanted islets. In addition, even though the transplanted islets are generated from patients’ own iPSCs (Thus MHC matching), long-term immune suppression may be still required due to autoimmune reaction against insulin in T1D patients who are already presented insulin as an antigen. However, some of these immunosuppressive drugs can be toxic to transplanted islets as they are located in the hepatic portal vein, which exposes them to the immunosuppressive drugs near serum levels. Extensive research is now focusing on identifying less toxic immunosuppressive regimens, as well as optimal sites that would not expose the transplanted islets to high concentrations of immunosuppressive drugs. There are also indications that even in the absence of immunosuppressive drugs, the function and viability of transplanted islets decline (116). The exact mechanism that mediates this decline is currently unknown, and direct observation and evaluation of the transplanted islet is often very difficult because of the location of the transplanted islets.

IBMIR is a major factor contributing to the initial loss of islets after transplantation, and strategies are being explored to prevent or mitigate the damage caused by this inflammatory reaction. One particular strategy is focused on the use of low-molecular-weight heparin, which has been shown to decrease IBMIR in many *in vitro* and *in vivo* animal models (53). Other potential strategies include thrombin inhibitors, complement inhibitors, as well as other anti-inflammatory tissues such as human adipose-derived mesenchymal stem cells (hADSCs) (117). It has been shown that the co-transplantation of islets with hADSCs or factors secreted by hADSCs can ameliorate the immune response against transplanted islets (82, 118), however, only heparin is currently used in clinical settings (119). Once the islets settle into the peripheral veins, it is difficult to monitor the islets for functionality and potential damage. Successful islet transplantation is often monitored indirectly, *via* c-peptide and insulin production, whereas IBMIR is based on the measurement of thrombin-antithrombin complex levels. The reported decline in the function of transplanted islets, even in the absence of cytotoxic immunosuppressants is very difficult to monitor because of the nature of the transplantation sites, in which direct visualization is too invasive, and current technology for visualization cannot properly gauge the health and functionality of the engrafted islets. The proximity of the liver to the gastrointestinal tract also indicates that liver-transplanted islets are exposed to toxins, antigens, and metabolic products from the gastrointestinal tract and its resident microbiota. As such, changes in gut microbiota and gut barrier integrity can contribute to the functionality and survival of transplanted islets (53). Microbiota diversity and gut function are altered in T1D patients (120–122). The effects of a pre-existing diabetes-linked microbiome have not been explored in islet transplantation, but these are the factors that must be considered when assessing the survivability and functionality of transplanted islets.

All of these liver-associated factors that contribute to the decline of transplanted islets highlight the need for an extrahepatic site for islet transplantation that would be less toxic to the transplanted islets. Alternative transplantation sites and techniques that would allow for rapid observation and evaluation of the transplantation are needed.

### Anterior Eye Chamber Transplantation Site

The anterior chamber of the eye (ACE) has been gaining considerable interest as an alternative site for islet transplantation because of its accessibility, highly vascularized oxygen supply, and immune-privileged character. A previous study demonstrated that in mouse and non-human primate models of T1D, intraocular islet transplantation showed superior efficacy and immune modulation that improved hyperglycemia more in the long-term than that of the liver transplantation site. Although clinical trials in humans are currently planned for patients with T1D who are legally blind in the eye where the islet is to be transplanted, it is not known how the graft islets in the ACE affect vision and related neural systems. This may limit the opportunity for intraocular islet transplantation in non-blind T1D patients. Nevertheless, this transplantation method is also beneficial for the non-invasive monitoring of graft survival. It has been demonstrated that the ACE allows for adequate engraftment of tissues from the heart, muscles, pituitary gland, liver and prostate (123–125). The dense vascularization of the iris allows for rapid angiogenesis of transplanted tissue contributing to the successful engraftment and survival of the graft (126). This site also offers the advantage of the graft being visualized non-invasively through the see-through cornea. The same advantages of ease of access, vascularization, innervation, and immune privilege also apply for pancreatic islet transplantation into the ACE.

Pancreatic islet transplantation into the ACE has been tested in rodents and non-human primates (NHP). The ease of access and straightforward surgical procedure allows relatively simple transplantation for monitoring of pancreatic islet’s functionality-coupled morphology in single islets or single-cell resolution (126, 127). In brief, the islets are carefully introduced to the cornea near the sclera through a small perforation, avoiding damage to the iris and bleeding. After which islets are allowed to settle into the cornea for approximately half an hour to facilitate attachment to the iris (127). The high vascularization density in the eye allows for rapid vascularization of transplanted islet. In mice, angiogenesis can be observed in as little as 24 hours (128), whereas complete vascularization is observed within four weeks at the same vascular density compared to native islets (127). Revascularization begins with the appearance of a large blood vessel followed by progressively smaller capillaries (70). The newly formed capillary network originates from a combination of endothelial cells within the transplanted islets as well as endothelial cells from the iris (67). Even in the absence of endothelial cells from the pseudo-islet or islet, vascularization can still occur with adequate fenestration that allows for the exchange of nutrients similar to that of native islets (67, 69).

The ACE also allows for reinnervation of the transplanted islets. Dense innervation in the eye contributes to the sympathetic and parasympathetic innervation of transplanted islets (124), which is important for the modulation of insulin release (129). Reinnervation starts at third days and plateaus at three months with an innervation pattern similar to that of native islets (66). As such, ACE engraftment has also been used to study innervation. It is important to note that the nerves innervating islets are probably not connected to the hypothalamic region but to a different central autonomic nervous circuitry compared to the native islets, affecting islet function modulation and not control (66). More research is needed to delineate the different effects of neuronal circuitry and their primary and secondary effects on glucose homeostasis (130). One major advantage of ACE transplantation is the relatively low number of islets required for complete control of hyperglycemia. In rodents, approximately 125 islets are sufficient to completely reverse diabetic symptoms and achieve normoglycemia, and only 50 islets are required to increase survival of rodent models of T2D (68, 126). Transplantation into the kidney capsule, a site often used as a control for islet transplantation in rodent, requires approximately 250 IEQ, which is double the number required to produce the same effect in the ACE (68). These remarkable effects have been attributed to increased and rapid vascularization and innervation. This reduced number of islets required to alleviate hyperglycemia is a major advantage of ACE transplantations that circumvent the limitations of shortage of islets and donors. Another contributing factor to the utility of the ACE for islet transplantation is the status of sites as immune-privileged at certain conditions, even allowing for immune-tolerance.

The ACE microenvironment is rich in immunosuppressive molecules that influence the activity of immune cells and the presentation of antigens. This phenomenon is termed as anterior chamber-associated immune deviation (ACAID), in which the aqueous microenvironment inhibits T-cell proliferation in mixed lymphocyte reactions and T-cell proliferation (131, 132) suppresses IFNγ production and promotes TGFβ (133). Immune-modulatory factors have also been identified in the ACE. These factors include αMSH and CGRP along with TGFβ, which suppresses the activation of inflammatory macrophages (134, 135); the MIF protein that prevents NK-cell activation, and the FasL protein that suppress activation of Th1 T cells and also regulates neutrophil and macrophage activation (136). There is also atypical and lower expression of MHC class I molecules and no expression of MHC class II molecules in corneal epithelial tissues is observed (137–139). Certain T cells are also inhibited and transformed from IFNγ-producing T cells to TGFβ-producing regulatory T cells.

The immune-modulating molecules in the ACE environment establish a barrier that maintains the induction of inflammation under control, even during transplantation. It is important to note that this immune-privileged status of the ACE is achieved only in the absence of a lymphatic bridge form connecting the ACE to the lymphatic system. Transplanted islets expressing MHC class II proteins as well as damage from surgical procedures can induce an immune response compromising the immunotolerant environment of the ACE (68, 135). Because of this, the ACE can be used to monitor auto-immune response in islet cells, while simultaneously monitoring the morphology of transplanted islet. A study by Tun et al. demonstrated that total of 12,000 islets (1500 IEQ/kg) transplantation in a left eye site in cynomolgus monkeys induced with early T2D through a high fat diet was able to delay progression of T2D and related complications (72). The authors observed that three months after immunosuppressants were withdrawn, the primates were still able to control hyperglycemia, indicating an acquired immune privilege/tolerant status for the transplanted islets. A similar study in 2011 by Perez et al. employed islet transplantation into the ACE of baboons using 20,000 IEQ (71). In both baboons and cynomolgus monkeys, glucose control was achieved with the transplanted islets without any clinically significant damage to the vision. However, innervation of islets in the eye may not be connected to the hypothalamic region of the brain, which controls glucose homeostasis. In addition, light has a potentiating effect on islets, potentially affecting insulin secretion and control (66). Finally, transplantation in the eye may result in the formation of cataracts, and collagen deposition into the eye blood vessels (70, 140). Anterior chamber of the eye is promising islet transplantation site, as demonstrated in different animal models including non-human primates but there are hesitations to its clinical applicability especially with the eye being a very sensitive and important organ. Transplantation into the anterior chamber of the eye demonstrated minimal islet required to achieve normoglycemia, coupled with robust vascularization and innervation post-transplantation, followed by immune-privileged microenvironment with the possibility of immune-tolerance being achieved. These are the ideal conditions for an islet transplantation site, but whether the same conditions can be achieved in humans is yet to be determined. Possible side effects such as cataract formation also need to be investigated. There are currently two recruiting clinical studies utilizing anterior chamber of the eye as islet transplantation site (NCT02916680 and NCT02846571). NCT02846571 is a pilot study aiming to transplant islet into severely visually impaired diabetic human eye to determine the safety of the procedure while NCT02916680 aims to determine the safety and efficacy of the procedure in healthy anterior chamber of the eye. These clinical studies would elucidate the applicability of the islet transplantation in the anterior chamber of the eye and whether the impressive results in animal models can be recapitulated in human patients.

### Bone Marrow Transplantation Site

Bone marrow is an alternative candidate for pancreatic islet transplantation because of its specific microenvironment. The bone marrow is an ideal site for islet transplantation because it is well-protected from external shocks, extravascular and well-vascularized. The presence of extensive vascularization without direct contact with blood, is essential for its functionality and viability. The broad distribution of bone marrow and ease of access allow multiple transplantations at different sites, overcoming the aforementioned size and amount limits. These limits impede hepatic portal vein transplantation due to constrains in portal vein pressure, and other similar technical and surgical restriction. Bone marrow transplantation is also a potentially less invasive procedure, with low risk and easy access for sampling *via* bone aspiration biopsy. However, a possible ramification of bone marrow transplantation is that hyperinsulinemia has the potential to contribute to hyperproliferative diseases such as bone marrow cancer development through the growth-promoting effects of elevated insulin. Yet this risk is also present in intraportal infusion as observed in some diabetic rats that received intraportal islet infusion and had an increased incidence of adenomas and hepatocellular carcinoma (141, 142).

In 2009, Cantarelli et al. used syngeneic mouse models for pancreatic islet transplantation into the bone marrow. In their model, the transplanted islets survived for more than a year without compromising hematopoietic activity with better metabolic parameters (73). The percentage of mice achieving normoglycemia and the timing were superior in bone marrow compared to intrahepatic infusion using the minimal mass model. Simultaneously, the quality of glucose metabolism in bone marrow was similar to that of intraportal infusion. The morphology and cellular composition of the bone marrow-transplanted islets showed significant changes, including increased size and a more compact morphology, which was attributed to the isolation and preparation method. The ratio of islet alpha cells to the beta cells in bone marrow transplant was also similar to islet control, whereas this ratio was decreased in intraportal infusion which is a possible explanation for the deficient glucagon response observed in many intrahepatic portal vein transplant patients.

As proof of concept, Cantarelli et al. performed autologous pancreatic islet transplantation into the iliac crest bone marrows of four human patients that had contraindication for intraportal infusion. This was the first report of successful endocrine tissue engraftment in the bone marrow (74). These patients underwent total pancreatectomy, and islet engraftment was successful based on the circulating c-peptide levels after islet transplantation into the bone marrow. All four patients required exogenous insulin treatment but maintained good glycemic control with sustained endogenous insulin production. Red and white blood cell levels and platelet counts were unaffected by the presence of islets in bone marrow and were within the normal, expected values. Bone marrow biopsies displayed the presence of all four types of pancreatic islet cells (insulin, glucagon, somatostatin, and pancreatic polypeptide) one year after transplantation as evidenced through histological staining and the quantitative PCR for mRNA markers of normal pancreatic development, function, and differentiation. The presence of CD34+ endothelial cells was indicative of islet neovascularization. Their report indicated that islet transplantation into the bone marrow was a safe and reproducible approach, with the bone microenvironment able to support islet revascularization and function. Their biopsy also indicated that transplanted islets can be monitored through a simple aspirate biopsy procedure because the bone marrow is enclosed. This is a major advantage over intraportal infusion, in which islet normally engrafts randomly in the hepatic portal vein capillary tree making it harder to monitor. However, this success in islet auto-transplantation into the bone marrow was not reinforced when the same researchers performed islet allotransplantation into the bone marrow of T1D patients. Their pilot randomized trial in 2019 with T1D patients showed graft loss in most of the patients within four months, independently of the induction agent or presence of maintenance immunosuppression. From their biopsies and antibody responses, the authors concluded that this rejection was a result of the autoimmunity recurrence and that this rapid rejection of pancreatic islet graft in the bone marrow may be caused in closer proximity to bone marrow tissue-resident mature CD4+ and CD8+ T cells. Furthermore, the bone marrow microenvironment post-transplantation of islets may contribute to the expansion of autoreactive T cells, such elevated concentrations of IL-7, or the low oxygen tension in the bone marrow despite very high vascular density. Finally, the authors concluded that the success seen in the pre-clinical and NHP models of T1D is difficult to replicate in humans without creating models of autoimmune-mediated rejection of pancreatic beta islets (75). Bone marrow as a transplantation site may not be ideal for pancreatic islet without first preconditioning the local bone marrow microenvironment for immune-modulation or cytoprotection with localized bone marrow irradiation (76)

### Subcutaneous Space Transplantation Site

Subcutaneous transplantation is a very attractive alternative site for islet transplantation, due to simplicity of the surgical procedure, unlikelihood of surgical complications, ease of access for graft monitoring, and possible retrieval of the transplant. However, the relative avascular nature of the subcutaneous space, and therefore lack of access to nutrients and oxygen hampers the utility of this transplantation site. As such, subcutaneous transplantations often requires the use of bioengineering devices and biomaterials, drug and trophic factors delivery systems and strategies to induce early angiogenesis, without which transplanted islets would not engraft sufficiently to achieve normoglycemia (143). There are various strategies in engineering biocompatible biomaterials that would induce vascularization and contribute to the survival and engraftment of transplanted islets. Numerous technologies encompassing bioengineering materials compatible with islet transplantation have been developed and tested (143–146). Biocompatible and biomimetic biomaterials such as hydrogels have been developed from Extracellular matrix (ECM)-based natural polymers such as collagen, fibronectin, fibrin, laminin, and alginate, and synthetic polymers such as polyethylene glycol (PEG), PGA, polyvinyl alcohol (PVA) and dextran. These biomaterials can be specifically designed to exhibit precise and tunable mechanical (stiffness), biological (incorporation of growth factors and bioactive cues) and biochemical (degradability, sensitivity to enzyme, cell adhesion) properties, to achieve a desired biological outcome. In addition, they can exhibit pro-angiogenic properties and promote the formation of vessels in the subcutaneous niche. Biomaterials can be engineered to accommodate not only pancreatic islets but also other cell types, such as hADSCs which can significantly improve transplantation outcomes. These biomaterials can be used to coat pancreatic islets, termed as micro/microencapsulation, or further developed into an implantable bulk scaffold that can allow for the exchange of nutrients throughout the hydrogel and to the encapsulated islets. Simultaneously hADSCs can act as a physical barrier to protect the islets from immune cells. Vlahos et al. developed a collagen-based hydrogel coated with endothelial cells (80). These collagen-based hydrogels were transplanted to create subcutaneous vascularized tissue implants. They showed that vascularization can be completed within 14-21 days and reverses hyperglycemia in approximately 10 days after transplantation. Another pro-angiogenic strategy they used focus on hADSCs and endothelial cells within collagen scaffolds. However, this study did not examine graft viability beyond 21 days. In 2015, Pepper et al. reported the development of a deviceless transplantation strategy for islet transplantation (78) in which they used biomedical-grade nylon catheters inserted subcutaneously and left for 30 days in order to create a transplantation site cavity in which they transplanted islets after removal of the catheters. There was no significant development of mature fibrotic scarring prior to implantation of islets by utilizing natural foreign body innate immune response. Using syngeneic, as well as immune-compromised mouse models of T1D, they showed that normoglycemia could be achieved in 91% of the mice for over 100 days using islets from both mice and humans. In contrast, Kim et al. on the other hand used esterified collagen along with heparin and hADSCs for their islet transplantation into the subcutaneous space of NOD mice (84) and found that this system improves normoglycemia better than with native collagen alone. Interestingly, they also showed that this effect is dosage-dependent on hADSCs, indicating that hADSCs or factors secreted by hADSCs is a contributing factor to survival and vascularization of transplanted islets. Yu et al. reported that the islet viability matrix (IVM) consisting of a mixture of collagen 1, L-Glutamine, fetal bovine serum, sodium bicarbonate and medium promotes islet survival for more than 150 days when transplanted subcutaneously in diabetic and immunodeficient mice (85). However, when applied to NHP models, the system failed to achieve euglycemia as they remained diabetic and required exogenous insulin despite the presence of insulin and glucagon positive islets and absence of fibrosis or mononuclear cell infiltration.

The skin is considered one of the largest organs in the body and one major advantage of subcutaneous transplantation is the potential availability of countless sites for transplantation. However, not all subcutaneous spaces present the same benefits as some might be more vascularized than others. Yasunami et al. performed subcutaneous islet transplantation in inguinal subcutaneous white adipose tissue, which has a feeding vessel from the inferior epigastric artery and vein (81). Their approach induced normoglycemia with 200 syngeneic islets (the number of islets that can be isolated from a single donor) equivalent in streptozotocin (STZ)-induced diabetic mice.

The subcutaneous site is an ideal transplantation site when combined with macro devices. Wang et al. demonstrated that a nanofiber-integrated (NICE) device enabled the reduction of the fibrotic response and allogenic response in FVB islet transplantation in C57BL/6J recipient mice (87). In addition, the NICE device protected 2,500 clusters of human pluripotent stem cell-derived functional β cells (sc-β cells) from xenograft rejection and achieved normal glycemia for more than 30 days in immune-competent C57BL/6J mice (87). Inadequate oxygenation at subcutaneous space is another factor limit the viability of transplanted islets. Recently, Wang et al. demonstrated that Inverse breathing Encapsulation Devise (iBED), a silicon-based gas exchangeable materials improves oxygen (O_2_) delivery is encapsulated islets and shows prolonged survival in multiple xenograft models (88). Challenge of transplantation of these islets with macro-and micro-encapsulation methods at subcutaneous sites still remain present as more islets (>2-5 times) are required to achieve normal glycemia with devices compared to naked transplantation.

Subcutaneous transplantation of islet remains very attractive when combined with angiogenic and immune-modulating biomaterials. However, clinical studies of such strategies have yet show definitive proof that islets can be sustained in long-term better than intraportal transplantation. The major reason for suboptimal performance is the subcutaneous immune response in which macro- and micro-encapsulated islets are often encased by fibrotic cells, cutting off the transplanted islet from oxygen and nutrition. Supposed immune-protective devices that physically separates the transplanted islets from immune cells, which underestimates the effect of diffusible immune factors on the functionality of islets. Such is the case for the device called Theracyte™ developed in the 1990s that utilized microencapsulated islets. The device was a sealed double-membrane device that show promise in murine studies but failed to achieve normoglycemia in higher mammalian models due to fibrotic overgrowth (147–150). A similar clinical study is currently ongoing utilizing Sernova cell pouch to create a subcutaneous microenvironment that can accommodate islet transplantation (NCT03513939). The patients will be in full systemic immune-suppression, indicating that immune-modulating function for subcutaneous transplantation of current devices still need further development.

Further investigation on advanced biomaterials with not only angiogenic ability but also immune-modulating capabilities for subcutaneous islet transplantation may be needed to achieve long-term islet survival.

### Omentum Transplantation Site

The omentum is a large, flat, thin adipose layer that hangs down from the stomach to cover the intra-organs (151). Due to the nature of superior neovascularization, tissue regeneration characteristics, hematostasis, and immune privilege, the omentum is considered an ideal nest for transplanted islets. Baidal et al. reported that islet transplantation in the omentum promoted long-term human islet survival and glycemic control in T1D patients and restored euglycemia and insulin independence more than 12 months (152). The trial is still ongoing and the data from longer term follow-up and recruiting more patients are required to assess long-term safety and efficacy of islet transplantation in omentum site.

The omentum can store a large amount of adipose tissue, including ADSCs; therefore, it provides a flat nest for immune cells, including macrophages, B-lymphocytes, T-lymphocytes, and mast cells, which wrap infection sites or wound tissues to protect vital organ activities (151, 153, 154). Although the omentum possesses a large number of immune cells, the immune responses of grafted islets here are lower than those of the subcutaneous sites (89). Weaver et al. demonstrated that in leukocyte density CD45+ or CD11b+ cells 4 weeks after mouse islet transplantation with a VEGF-conjugated hydrogel was significantly lower in the epidermal fat pad, which is considered a rodent omentum compared to that of the subcutaneous site in C57BL/6J mice (89). In addition, islets transplanted in the epidermal fat site have lower, controlled glucose levels compared to those transplanted at the subcutaneous site (89). Bochenek et al. demonstrated that alginate encapsulated islets transplanted into the omentum bursa of macaques are protected from allogenic rejection and sustain glucose responsiveness more than 4 months (92). With its extensive vascularization, immune modulation, easy accessibility, and non-vital site status, the omentum is considered one of the most attractive islet transplantation sites.

**Figure 2:**
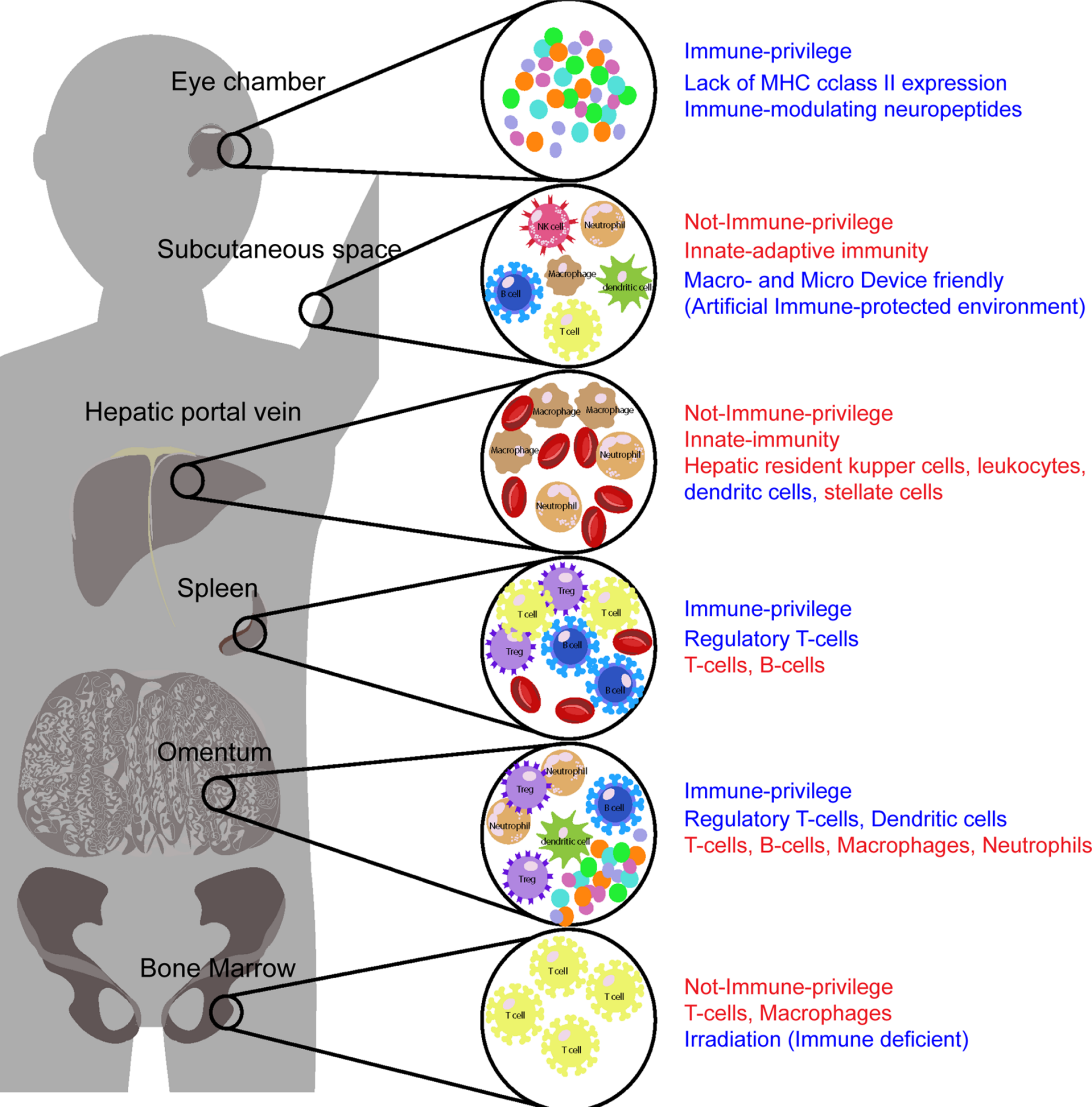
Major immune components in each site affecting success of transplantation. Each islet transplantation sites have different microenvironment, which include immune system components that either increase or decrease of islet engraftment. Some of these sites, such as the omentum, spleen and anterior chamber of the eye, are considered immune-privileged. The presence of anti-inflammatory factors and regulatory T-cells that suppress expansion of effector T-cells and decreasing the effects of proinflammatory cytokines are key immune modulators in immune privilege sites. Other sites, such as the subcutaneous space and the bone marrow sites contain T cells and NK cells, among other immune cells that contribute to graft rejection and long-term viability of transplanted islets. Strategies must be incorporated in these sites to not only increase angiogenesis but also modulate immune responses to prevent graft rejection. The potential anti-inflammatory components in each site are described by blue color and the potential inflammatory components in each site are described by red color.

Ultimately, the goal of islet transplantation research using the omentum site is to restore insulin production in diabetes patients without the need for immunosuppressive drugs. Further study is needed to investigate whether the omentum can promote long-term survival and safety of stem cell-derived islets (43, 155). The comparison of immune cell contribution in each transplantation sites include omentum are shown in **Figure 2**.

### Spleen Transplantation Sites

Spleen is a peripheral lymphoid organ that has functions for maturation of adaptive immune cells including B cells, T cells, plasma cells and production of immunoglobulin. It is highly vascularized and drains into the portal venous system, making it a potential candidate nest for islet transplantation. Spleen is involved in the regulation of autoimmunity, as well as in the suppression of T cell proliferation by induction of immune tolerance. In a study by Ryu et al. in 2001 demonstrated that normal splenocytes with partially or fully matched for MHC class I antigens restores self-tolerance and eliminates β-cell directed autoimmunity in NOD mice (156, 157). Spleen also contains Regulatory T-cells (Tregs) that suppress T cell proliferation, as well as dendritic cells that secrete suppressor cytokines including TGF-β, IL-10, and IL-35 which further contributes to the induction of immune tolerance (158–161). These characteristics of spleen therefore could potentially contribute to the better engraftment and long-term survival of islet transplants.

Despite above advantages, islet transplantation in the spleen sites is still controversial. A study by Stokes et al. in 2017 showed that porcine islet transplantation into the pig spleen is not significantly better than transplantation into the kidney capsule or liver, rather it is inferior to the kidney capsule according to the glucose tolerance test (GTT) results (95). Similar results were obtained when the same authors performed transplantation experiments of murine and human islets unto murine subjects at different transplantation sites - their results showed poor engraftment into the spleen, when compared to portal vein transplantation (96). And yet, a study by Itoh et al, in 2017 showed that minimum mass of syngeneic islet needed for transplantation into spleen splenic pulp is around 50 IEQ, compared to 200 IEQ in portal vein, and 1,000 IEQ in kidney capsule is needed to mitigate hyperglycemia, indicating that transplantation into the spleen can be achieved and at certain conditions, can function better than transplantation into intraportal vein or kidney (94). This contradiction in experimental outcomes makes it hard to assess the utility of spleen as an islet transplantation site. However, an explanation to the outcome differences may be from differences in surgical procedure and transplantation techniques wherein islet engraftment can be surmised to have low efficiency if islets are introduced to the spleen *via* the blood vessels or through the pulp since the environment would contain red blood cells and coagulation factors similar to hepatic portal vein and thus would induce IBMIR and lead to islet loss. Avoiding damaging the spleen and minimizing islet contact with the blood by transplantation into spleen subcapsular space may increase the engraftment efficiency but this has yet to be demonstrated in higher mammalian models (162). The difficulty of the surgical procedure and limited subcapsular space available make transplantation into the spleen challenging. Nevertheless, advancement in surgical techniques can expand the potential of spleen as a transplantation site.

The spleen is considered as not only nest for islet transplantation but also as a source of stem cells that can be differentiate into insulin producing cell which may have utility in regeneration of pancreatic β cells (163, 164). A study by Kodama et al. in 2003 showed that CD45- splenocytes can develop into stem cells and further differentiate into islet progenitor cells (156). A study by Itoh et al. in 2017 further provide evidence that at islet graft expansion can occur when transplanted in the spleen by transplanting 25 islets into the spleen along with 100 islets in the kidney. The islet number transplanted into the spleen that is normally incapable of achieving normoglycemia. However, after 240 days, eight out of eleven mice maintained normoglycemia despite nephrectomy (94). More than just a transplantation site, spleen may therefore help with the survival, regeneration and expansion of insulin producing cells.

### Intramuscular Transplantation Site

The muscles have been used as transplantation site for many decades, specifically for auto-transplantation of parathyroid glands. Auto-transplantation of parathyroid cells in between muscle fibers have been shown to have successful long-term outcomes with minimal side effects, prompting consideration of muscles as a potential transplantation site for islets.

Intramuscular transplantation offers several advantages. First, the muscles are capable of forming dense vasculature such as during exercise, with oxygen tension reaching values close to that of native pancreas. Since hypoxia and angiogenesis have been major factors affecting success of engraftment of islets, this ability to form extensive vasculature is beneficial not only for early engraftment and survival of transplanted islets but also in its long-term functionality and responsiveness to blood glucose. Secondly, surgical procedure of intramuscular implantation is relatively easy, can be done under local anesthesia with minor risks and complications. Transplantation into the muscle interstitium would minimize contact of islets with the blood and avoid IBMIR-related islet loss. Furthermore, intramuscular transplantation would allow easy retrieval, biopsy, and monitoring of engrafted islet which is a major advantage over most transplantation sites where monitoring and retrieval have been complicated by potential major tissue damage. Thirdly, islets can be transplanted in multiple muscle sites and therefore allow for multiple and repeated implantation and explantation of transplant tissue.

The muscle is therefore a very attractive site for islet transplantation but the challenge is still remaining. Similar to subcutaneous tissues without neovascularization pretreatment, the muscle microenvironment is hypoxic and early transplantation studies could not achieve normoglycemia or long term normoglycemia (165–168). Similarly, the transplantation efficacy in the muscles have been shown to be worse than that of kidney or liver (102). It is estimated that lack of neovascularization contributes greatly to the poor outcome of transplantation in the muscles. Just like subcutaneous transplantation, it is generally required for the muscle to be pre-treated to induced neovascularization before islet transplantation, with angiogenic factors and biocompatible devices. Witkowski et al. demonstrated that two weeks prior pre-treatment of alginate-based bio-scaffold containing angiogenic factors and extracellular matrix peptide motif RGD in the abdominal musculature of male Lewis rats sustained synergistic islet survival upto 60 days until removal of the islets (100). Similar experiments have been done by Tsuchiya et al, using matrigel to improve islet survival and muscular vascularization. In both of these studies, syngeneic islets were used which precludes another major factor in intramuscular formation which is the occurrence of extensive fibrosis and immune response (105). The muscle microenvironment is not immune-privileged and in the first few hours after transplantation, myocyte-derived pro-inflammatory cytokines such as IL6, IL8 and MCP-1 can be detected (169, 170). Pro-inflammatory cytokines are known to affect islet engraftment failure by contributing to early islet central necrosis and fibrosis. While fibrosis can be reduced by careful transplantation and pearl-on-a-string arrangement of islets to avoid aggregation, this is insufficient to bring the engraftment efficiency comparable to intraportal transplantation (101).

Muscle is metabolically active and contractile organ. During exercise and similar activity, the muscle consume glucose and produce lactate. It is not yet fully investigated how anabolic and catabolic requirements of muscle might affect transplanted islets and their function (171). Similarly muscle contraction can affect blood supply and further negatively affect the function of transplanted islets. In this regard, Kim et al. investigated how denervation and creation of gastrocnemius muscle flap might be beneficial for intramuscular islet transplantation (108).

Intramuscular transplantation of islets have been investigated for clinical application, and the most notable was the auto-transplantation into the brachioradialis muscle in a 7-year old patient with contraindication for liver intraportal transplantation. The transplanted islet were able to survive and the transplantation site was considered safe without any significant surgical or post-surgical complications proving the site’s advantages and utility for graft monitoring and possible explantation (99). The patient was able to achieve an improvement in quality of life and glycemic control for two years although insulin independence was not achieved. In another study, four patients in which liver intraportal allo-transplantation was a contraindication, allogeneic human islets were transplanted into the brachioradialis forearm muscles. While there are no surgical complications, the transplanted islets were progressively became nonfunctional indicating the challenges of intra-muscular transplantation especially for allogeneic tissues (107). There is one clinical study currently ongoing in which islet, along with autologous mesenchymal stem cells are transplanted into the forearm intramuscular tissue of patients that are also undergoing kidney transplantation, to determine the immunomodulating effects of mesenchymal stem cells that could help sustain long term viability of intramuscularly transplanted islets (NCT01967186). These clinical studies highlight the need for research focusing on strategies not only to induce early neo-angiogenesis but also to minimize immune response to intramuscularly transplanted islet. We have demonstrated that stem cell-derived pancreatic organoids that are not only capable of secreting insulin in response to varying glucose concentration but are also immune evasive by modulating PD-L1 expression (43). Similarly, bio-compatible hydrogels and scaffold offer promising strategies such as a PD-L1-presenting biomaterial by Coronel et al. in which polyethylene glycol hydrogel are engineered to contain the T-cell immunomodulating protein PD-L1. While this study still make use of rapamycin in combination with the PD-L1-containing hydrogel, it shows the potential of bio-compatible materials to be developed to contain immune-modulating properties. Intramuscular transplantation of islets, similar to subcutaneous space, remains an attractive option due to the site’s advantages of accessibility and vascularization potential but its application is hampered by the robust immune response and short life span of allogeneic islets. Combining with further precise control of local β cell inflammatory responses (172), islet graft survival may be enhanced. Bio-compatible materials and immune-modulating strategies may be able to overcome these limitations. Its similarity with subcutaneous space in terms of advantages but further limited by the undetermined effects of muscle microenvironment during active metabolism makes intra-muscular transplantation a secondary choice to subcutaneous space.

## Conclusions

Current advances the field of islet transplantation for the treatment of diabetes focus on investigating the microenvironment of transplantation sites, including their vascularization, extracellular matrix content, and tissue-resident immune cells. To date, no consensus was reached on identifying an ideal optimal islet transplantation site. An increasing amount of research is now focusing on developing engineered hydrogel-based materials and macrodevices in order to create a transplantation space, protect the graft for the immune system, and promote angiogenesis. Although current advances on biomaterial approach for islet transplantation, the challenges remain to achieve normal glycemia with the optimal amounts of cadaveric human islets or human stem cell-derived islets. Each transplantation sites still lacks the ability to accommodate the long-term survival of islets. In particular, it is unclear whether aging and other physiological health conditions influence the survival of transplanted islets. In addition, although the current advances on stem cell technologies and genetic manipulation enable us to generate functional stem cell derived islets, long-term efficacy of biomaterials for encapsulating these artificial islets remains unknown. In addition, the risk of post-implantation trans-differentiation, teratoma formation, or graft invasion to other sites needs to be carefully investigated. Improving islet graft survival, engraftment, and efficacy in highly vascularized, nutrition and an oxygen-rich and immune-regulated sites remain the priority to improve long-term efficacy and safety for islet cell therapy to improve the outcomes of diabetic patients.

## Reference styles

Science, Engineering and Humanities and Social Sciences references.

## Author Contributions

FC, LN, and EY wrote and edited the manuscript. EY conceptualized and obtained funding for this study. All authors contributed to the article and approved the submitted version.

## Funding

This work was supported by the funding from California Institute for Regenerative Medicine (CIRM)-DISC2 discovery award, Integrated Islet Distribution Program (IIDP) Pilot award, Allen Foundation grant, Mishima Kaiun Memorial Foundation Research award, Lundquist Institute Voucher award and CTSI-UCLA awards.

## Conflict of Interest

EY is inventor on licensed patents and patent applications related to the HILOs technology described in this manuscript.

The remaining authors declare that the research was conducted in the absence of any commercial or financial relationships that could be construed as a potential conflict of interest.

## Publisher’s Note

All claims expressed in this article are solely those of the authors and do not necessarily represent those of their affiliated organizations, or those of the publisher, the editors and the reviewers. Any product that may be evaluated in this article, or claim that may be made by its manufacturer, is not guaranteed or endorsed by the publisher.
